# The influence of physical exercise on college students’ prosocial behavior: the chain mediating role of social conscientiousness and general self-efficacy

**DOI:** 10.3389/fpsyg.2026.1813198

**Published:** 2026-04-22

**Authors:** Guoqiang Song, Xingyu Yi, Yuandai Chen

**Affiliations:** 1 Physical Education Institute, Hanjiang Normal University, Shiyan, China; 2 Physical Education Institute, Hubei University Automotive Technology, Shiyan, China

**Keywords:** chain mediation, general self-efficacy, physical exercise, prosocial behavior, social conscientiousness

## Abstract

**Purpose:**

This study investigates the relationships among physical exercise (PE), social conscientiousness (SC), general self-efficacy (GSE), and prosocial behavior (PB) in college students.

**Methods:**

A scale was administered to 409 college students, and statistical analyses were performed using SPSS 28.0 and Process 4.1.

**Results:**

(1) Significant positive correlations exist among college students’ physical exercise, social conscientiousness, general self-efficacy, and prosocial behavior. (2) Physical exercise positively predicts college students’ prosocial behavior (*β* = 0.174, *p* < 0.001). (3) The effect value of indirect path 1 (PE → SC → PB) was 0.009, representing 38.9% of the total effect; the effect value of indirect path 2 (PE → GSE → PB) was 0.004, accounting for 16.9% of the total effect; and the effect value of indirect path 3 (PE → SC → GSE → PB) was 0.002, constituting 10.4% of the total effect.

**Conclusion:**

Physical exercise directly influences college students’ prosocial behavior and also exerts an indirect effect through social conscientiousness and general self-efficacy.

## 1 Introduction

The World Health Organization (WHO) explicitly states in the “Guidelines on Physical Activity and Sedentary Behavior” that physical activity not only enhances physical and mental health but also yields positive outcomes for social development, including the improvement of interpersonal relationships and a reduction in problematic behaviors (García-García et al., 2023). As a vital means of regulating both physical and mental health, as well as facilitating social adaptation, physical exercise is intricately linked to the cultivation of prosocial behavior and has become an essential internal requirement for both national and individual development (Chongjin et al., 2025). Prosocial behavior, a significant indicator of the advancement of modern social civilization, encompasses actions that individuals undertake in interpersonal interactions that benefit others, groups, or society. Its characteristics include helping, cooperation, sharing, and altruism (Feng and Xu, 2026). A review of the relevant literature reveals that while empirical research on the impact of physical exercise on prosocial behavior is gradually expanding, existing studies seldom investigate the synergistic effects of personality traits and cognitive factors. Consequently, the mediating pathways and influencing mechanisms remain inadequately explored (Wan et al., 2021; Jiang and Bian, 2025b).

Based on the interactive framework of “personality → cognition → behavior” in social cognitive theory, which posits that the occurrence of individual behavior is influenced by the external environment and regulated by individual cognition and personality traits (Hirschbeck et al., 2024), this paper selects social conscientiousness and self-efficacy as mediating factors. Social conscientiousness is an important dimension of personality traits, referring to an individual’s tendency in goal setting, plan execution, self-restraint, and sense of responsibility. Its core connotations are being responsible, reliable, abiding by norms, and fulfilling commitments (Huang et al., 2025). It is closely related to but fundamentally different from prosocial behavior: social conscientiousness focuses on self-regulation during the behavior process, while prosocial behavior centers on altruism (García-García et al., 2023). As an individual’s belief in their own abilities, self-efficacy is an important evaluation indicator of self-cognition. In summary, combining social cognitive theory with existing research gaps, this paper proposes the following hypotheses regarding the relationships among physical exercise, social conscientiousness, self-efficacy, and prosocial behavior.

## 2 Research hypotheses

### 2.1 Predictive effect of physical exercise on prosocial behavior of college students

Physical exercise refers to physical activities that utilize various sports methods in combination with natural forces (sunlight, air, and water) and health measures, aiming to develop the body, improve health, enhance physical fitness, and bring pleasure to the mind and body (Han et al., 2025). It plays a crucial role in promoting human growth and development, shaping a healthy and beautiful physique, improving the body’s working capacity, regulating emotions, and enhancing people’s quality of life (Guo et al., 2023). Relevant research shows that there is a significant positive correlation between physical exercise and prosocial behavior (Jiang and Bian, 2025). First, at the physiological level, physical exercise stimulates the brain to produce “oxytocin” and the body to produce “endorphins,” which are known as the “love hormone” and the “moral factor” (Salleg-Cabarcas et al., 2024). They can enhance trust and empathy between people, act as a catalyst for a pleasant mind and body and anxiety reduction, and make individuals more likely to show kindness and patience, thus providing a favorable emotional background for prosocial behavior (Golaszewski et al., 2022). Second, sports participants abide by rules, train on time, and fulfill their responsibilities in the team, which strengthens individual responsibility and reliability and enhances the collective sense of honor, creating favorable conditions for prosocial behavior (Gibson et al., 2024). Finally, (Tang et al., 2025) also confirmed that college students who actively participate in sports activities tend to exhibit more significant prosocial behavior tendencies and less aggressive behavior. Based on the above, there is a correlation between physical exercise and prosocial behavior. This paper proposes Hypothesis H1: Physical exercise can positively predict the prosocial behavior of college students.

### 2.2 Mediating role of social conscientiousness

Social conscientiousness is derived from the “conscientiousness” trait in personality psychology. Individuals with high conscientiousness often exhibit characteristics such as being meticulous, having good planning, being well-organized, and having strong self-control (Ma et al., 2025). As a structured social situation, physical exercise gradually internalizes external social norms into individuals’ stable personality traits (social conscientiousness) through repeated rule-following, responsibility fulfillment, and goal achievement. This internalized Social Conscientiousness constantly drives the occurrence of prosocial behaviors (Tang et al., 2025). Additionally, as a “field for practicing responsibility,” physical exercise is an important place for shaping personality traits and cultivating problem-solving abilities through rule reinforcement, role division, and goal cohesion (de Bruijn and Brocken, 2025). These reliable and norm-abiding qualities will gradually transfer to social situations, resulting in behaviors such as helping others and being willing to share. Existing studies have confirmed that individuals with high conscientiousness are more inclined to abide by social norms and are more willing to participate in behaviors such as helping others, cooperation, and volunteer activities (Sabo et al., 2020). Other studies have also confirmed that people with high sense of responsibility are good at putting collective interests above personal interests and are more active in practical actions (Tang et al., 2025). Based on the above arguments, this study proposes Hypothesis H2: Social conscientiousness plays a mediating role between physical exercise and college students’ prosocial behaviors.

### 2.3 Mediating role of general self-efficacy

Self-efficacy, proposed by Albert Bandura, refers to an individual’s confidence in whether they can achieve a certain goal or complete a certain task. It does not refer to the actual ability but a prediction of one’s own ability, which often determines a person’s executive ability and perseverance (Bingxue and Yuzhong, 2025). When an individual adheres to exercise, overcomes physical inertia, and achieves exercise goals, they will gain a sense of success of “I can do it” (An et al., 2024). This confidence in one’s own ability will extend to other fields and form a belief with subjective initiative. Individuals with high self-efficacy are more likely to engage in prosocial behaviors because they believe they have the ability to help others and are not afraid of failure and setbacks. In addition, they are more willing to participate in complex social situations, believing that they can bring about positive changes. Some studies suggest that regular physical exercise can increase people’s self-confidence, reduce anxiety, and help them overcome timidity calmly (Guo et al., 2023). Other studies have confirmed that groups with high self-efficacy are more likely to help others and are willing to cooperate (An et al., 2024). Therefore, this study proposes the research hypothesis H3: Self-efficacy plays a mediating role between physical exercise and prosocial behaviors.

### 2.4 Chain mediating effect of social conscientiousness and general self-efficacy

**Figure 1:**
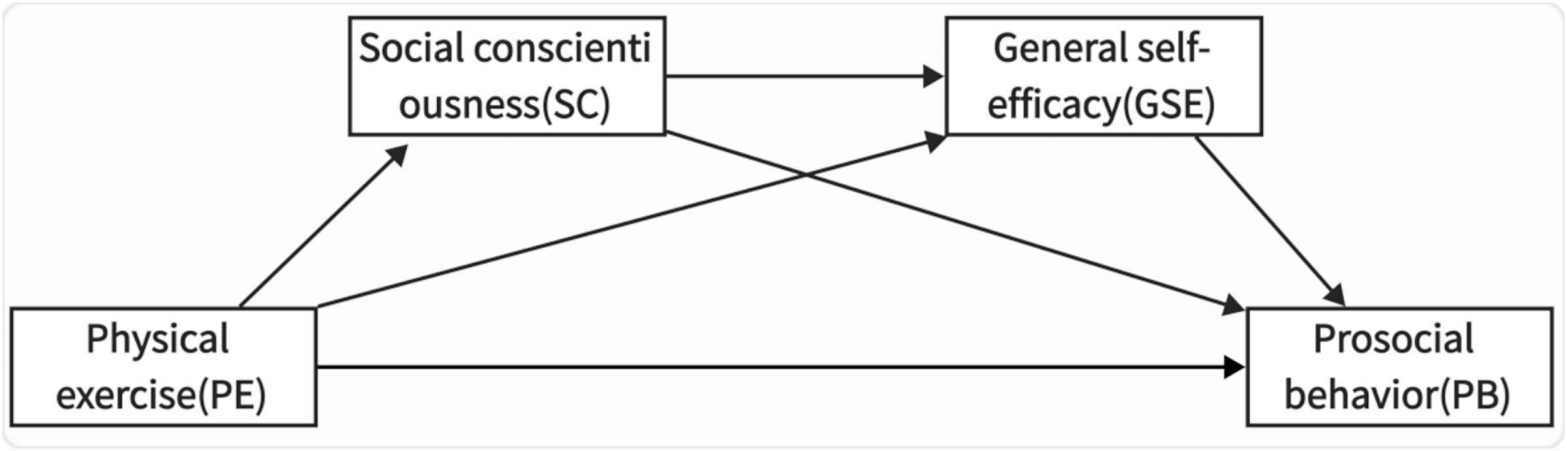
Schematic diagram of the hypothetical model.

In the influence of physical exercise on the prosocial behavior of college students, there is an interactive relationship between social conscientiousness and general self-efficacy. Studies by Ma et al. (2025) and Sánchez-Miguel et al. (2025) have shown that an increase in the level of conscientiousness can significantly affect self-efficacy, and individuals often show a tendency to be helpful. On the one hand, a high-conscientiousness environment is often accompanied by positive encouragement and feedback. Verbal and behavioral hints from peers, teachers, or family members provide a psychological support of “I can do it,” which will be internalized as individuals’ self-recognition (Guo, K. L. et al., 2025; Ma et al., 2025). On the other hand, people with high conscientiousness are more likely to have successful experiences. They reduce the uncertainty of the environment and the internal consumption psychology. This kind of positive vicarious experience is a source of increasing individual confidence (Gong et al., 2025). In addition, a study by Jiang et al. (2025) pointed out that people with high self-efficacy are more willing to help others. Helping others is often accompanied by risks and uncertainties. People with high self-efficacy have a high sense of behavioral control and believe that they have the ability to cope with these risks and uncertainties (Chongjin et al., 2025). Therefore, this study proposes Hypothesis H4: Social conscientiousness and general self-efficacy play a chain mediating role in the influence of physical exercise on college students’ prosocial behavior. This is in line with the construction of the external social environment and the cultivation of internal psychological capital, revealing the internal mechanism by which individuals achieve social growth through physical activities. The hypothesized model is shown in Figure 1.

## 3 Research objectives and methods

### 3.1 Research subjects

The research object of this paper is the influence of physical exercise on the prosocial behavior of college students, as well as the mediating roles played by social conscientiousness and general self-efficacy. To improve the representativeness of the sample, a random stratified sampling method was used to select college students from Hubei and Henan provinces (with moderate regional economic conditions and located in the central region) as the survey sample. Then, according to different urban development levels and discipline settings, two universities in provincial capitals were selected, namely Zhengzhou University (a comprehensive university in Zhengzhou) and Wuhan Business University (a finance and economics university in Wuhan), and two universities in non-provincial capital cities were selected, namely Hanjiang Normal University (a normal university in Shiyan) and Henan University of Chinese Medicine (a medical university in Xinxiang). Then, 438 online questionnaires were randomly distributed according to the ratio of current students (about 1,000:4). Among them, 29 invalid questionnaires were excluded. Invalid questionnaires included those with too short response time, repeated IP addresses, incomplete responses, and consecutive identical answers. The remaining 409 questionnaires were valid, with an effective recovery rate of 93.38%. The demographic information is shown in Table 1.

**Table 1:** Distribution of demographic variables among the survey respondents.

Causality	From	Quantities	Percentage
Gender	Male	199	48.7%
	Female	210	51.3%
Grade	1	94	23.0%
	2	95	23.2%
	3	102	24.9%
	4	118	28.9%

### 3.2 Measurement tools

#### 3.2.1 Physical activity rating scale

This study employed the Physical Activity Rating Scale (PARS-3) revised by Deqing (1994) to investigate the physical activity volume of the subjects in the past month. The scale consists of three dimensions: exercise intensity, exercise duration, and exercise frequency. Each dimension uses a 5-point Likert scale. The calculation formula for the physical exercise volume is: Physical exercise volume = Exercise intensity × (Exercise duration − 1) × Exercise frequency (Deqing, 1994). Based on the classification criterion that “moderate-intensity physical exercise can most effectively alleviate stress” (Deqing, 1994), the physical activity volume was divided into three levels: low volume (≤19 points), moderate volume (20–42 points), and high volume (≥43 points). In this study, the scale demonstrated good internal consistency, with a Cronbach’s *α* coefficient of 0.829, and also exhibited favorable structural validity, with a KMO value of 0.722 and *p* < 0.001 (Xinyi et al., 2026).

#### 3.2.2 Prosocial behavior scale

The Prosocial Tendencies Measure (PTM) developed by Carlo and Randall (2002) and revised by Yu et al. (Shi et al., 2026) was used to assess the prosocial behavior of college students. The scale consists of 23 items and 6 dimensions, namely public, anonymous, emergency, compliant, emotional, and altruistic, and is evaluated using a 5-point Likert scale. Higher scores indicate a stronger tendency for prosocial behavior. The overall Cronbach’s α coefficient of this scale is 0.943. Confirmatory factor analysis results indicated acceptable fit indices: *X*^2^/df = 4.870, RMSEA = 0.063, RFI = 0.906, TLI = 0.924. These results indicate that the PTM demonstrated good reliability and validity in this study.

#### 3.2.3 Social conscientiousness scale

The short-form version of the Chinese Big Five Personality Inventory was selected. This questionnaire was developed by Mengcheng et al. (2011). Five questions related to social conscientiousness in the questionnaire were chosen. These questions can fully reflect the connotation of social conscientiousness, and their reliability and validity have been verified. The internal consistency reliability of the questionnaire is 0.859, and the loading factors of each question range from 0.577 to 0.756. For the measurement model fit: *X*^2^/df = 14.304, CFI = 0.984, TFI = 0.972, RMSEA = 0.080, SRMR = 0.021, indicating good structural validity (Xianguo et al., 2024).

#### 3.2.4 General self-efficacy scale

The General Self-Efficacy Scale revised by Chinese scholars Wang Caikang and Zhongjun (2001) was adopted. Research has confirmed that this scale has high reliability and validity for measuring the self-efficacy level of Chinese college students (Niu et al., 2025). The scale consists of a 5-point Likert scale. The higher the score, the higher the individual’s confidence in accomplishing something. The overall Cronbach’s *α* coefficient of the scale is 0.962. In the exploratory factor analysis, KMO = 0.977, *p* < 0.05. The confirmatory factor indicators are as follows: *X*^2^/df = 3.465, GFI = 0.965, NFI = 0.974, IFI = 0.981, TLI = 0.976, CFI = 0.981, RMSEA = 0.053, indicating that the scale has good reliability and validity.

### 3.3 Data analysis

First, Excel and SPSS 26.0 software were used to organize and screen the questionnaire data, and invalid questionnaires were excluded. Then, Harman’s one-way test was employed to verify whether there was collinearity bias in the data. Next, analysis of variance was used to conduct difference tests with gender and grade as subgroups. Subsequently, Pearson correlation analysis was used to explore the correlations between variables. Finally, to verify the validity of the mediation model, the Process plug-in (Model 4) was selected for mediation effect testing, and the analysis was mainly based on the bias-corrected confidence intervals provided by the Bootstrap method (5,000 repetitions). This method is widely adopted in the field because it is suitable for small and medium-sized sample sizes and the analysis results are intuitive and robust (Huang et al., 2025).

### 3.4 Data collection

A questionnaire survey was conducted among college students in Hubei and Henan regions using cluster random sampling for an online questionnaire survey. The questionnaires were distributed on October 10, 2025, and all were collected and sorted out by November 3, 2025. The study was approved by the Ethics Committee of Hubei University of Automotive Technology (2026LLSC01). The study design adhered to the provisions of the Declaration of Helsinki, and all participants were informed in writing or orally and gave their informed consent.

## 4 Results and analysis

### 4.1 Bias testing of commonly used methods

Due to the influence of factors such as measurement conditions, subject characteristics, and cultural backgrounds, the data obtained from the questionnaire may have the problem of common method bias (Han et al., 2025). A Harman single-factor test was conducted on the measurement data. SPSS 29.0 was used to perform a rotated principal component analysis on physical exercise, social conscientiousness, self-efficacy, and prosocial behavior. The results showed that there were 4 factors with eigenvalues greater than 1, and the explanatory rate of the first factor was 23.458%, which was less than the critical value of 40%. This indicates that there is no common method bias problem in this study.

### 4.2 Descriptive statistics and correlation analysis of each variable

Descriptive statistical analysis shows (Table 1) that there are 409 college students in the sample, including 199 male students and 210 female students. This indicates that the number of male and female students participating in the survey is approximately equal, thus avoiding confounding bias. In terms of grade distribution, the senior students have the largest number, with 118 students, followed by 102 junior students, 95 sophomore students, and 94 freshmen. The sample size distribution among different grades is at an equal level.

ANOVA analysis was used to analyze the differences in four factors, namely physical exercise, prosocial behavior, social conscientiousness, and general self-efficacy, between genders and grades. The results showed that in the gender subgroup, there was a significant difference in physical exercise (*p* = 0.001), with boys having a higher level of physical exercise than girls. There were no significant differences in prosocial behavior, social conscientiousness, and general self-efficacy (*p* > 0.05). In the grade subgroup, there were significant differences in physical exercise and general self-efficacy (*p* = 0.001), with the third-grade students having higher levels than those in other grades. There were no significant differences in prosocial behavior and social conscientiousness (*p* > 0.05) (See Table 2).

**Table 2:** One-way ANOVA.

Category	*N*	PE	PB	SC	GSE
Sex: male	199	23.432 ± 22.345	3.694 ± 0.837	2.812 ± 0.778	2.790 ± 0.715
Female	210	21.642 ± 21.475	3.474 ± 0.849	3.267 ± 0.783	2.555 ± 0.777
F		41.293	0.874	0.921	4.076
P		0.001	0.350	0.338	0.154
Grade:1	94	21.147 ± 23.551	3.804 ± 0.754	2.782 ± 0.791	2.771 ± 0.511
2	95	19.643 ± 18.769	3.392 ± 0.879	3.686 ± 0.778	2.341 ± 0.818
3	102	21.712 ± 20.246	3.715 ± 0.911	2.712 ± 0.785	2.819 ± 0.784
4	118	17.663 ± 0.18.194	3.579 ± 0.806	2.742 ± 0.784	2.653 ± 0.728
F		9.305	3.416	3.382	7.670
P		0.001	0.116	0.118	0.001

As shown in Table 3, a Person correlation analysis was conducted on the data of physical exercise, prosocial behavior, social conscientiousness, and general self-efficacy. It was found that physical exercise had a significant positive correlation with prosocial behavior (*r* = 0.447), social conscientiousness (*r* = 0.446), and general self-efficacy (*r* = 0.472) (*p* < 0.01). Prosocial behavior had a significant correlation with social conscientiousness (*r* = 0.548) and general self-efficacy (*r* = 0.510) (*p* < 0.01), and social conscientiousness had a significant correlation with general self-efficacy (*r* = 0.499) (*p* < 0.01). This paves the way for subsequent structural equation and mediation analysis, indicating that the data meet the requirements of mediation analysis (Gerland and Baumann, 2024).

**Table 3:** Pearson correlation analysis.

Variables	PE	PB	SC	GSE
PE	1			
PB	0.447**	1		
SC	0.446**	0.548**	1	
GSE	0.472**	0.510**	0.499**	1

**p < 0.01.

### 4.3 Mediation effect test

Based on the hypothetical model, a regression analysis of the variables was conducted (Table 4). Taking physical exercise as the independent variable and social conscientiousness as the dependent variable, the standardized coefficient was found to be *β* = 0.447, *p* < 0.001. Therefore, there is a positive correlation between physical exercise and social conscientiousness, that is, for every one-standard-deviation increase in physical exercise, prosocial behavior will increase by one standard deviation. A step-by-step regression analysis was performed with general self-efficacy and prosocial behavior as the dependent variables. The results showed that physical exercise can positively predict general self-efficacy (*β* = 0.311, *p* < 0.001), and social conscientiousness can also positively predict general self-efficacy (*β* = 0.360, *p* < 0.001); physical exercise can positively predict college students’ prosocial behavior (*β* = 0.174, *p* < 0.001), social conscientiousness can positively predict college students’ prosocial behavior (*β* = 0.342, *p* < 0.001), and general self-efficacy can positively predict college students’ prosocial behavior (*β* = 0.257, *p* < 0.001). This result provides support for the mediating effect test.

**Table 4:** Regression analysis among variables.

Equation of regression	Variable of prediction	Overall fit index			Significance of regression coefficients		
Result variable		*R*	*R* ^2^	*F*	*β*	*t*	*p*
SC	PE	0.447	0.200	101.841	0.447	10.092	0.000
GSE	PE	0.572	0.327	98.524	0.311	6.845	0.000
	SC				0.360	7.919	0.000
PS	PE	0.629	0.396	88.567	0.174	3.809	0.000
	SC				0.342	7.389	0.000
	GSE				0.257	5.456	0.000

**Figure 2:**
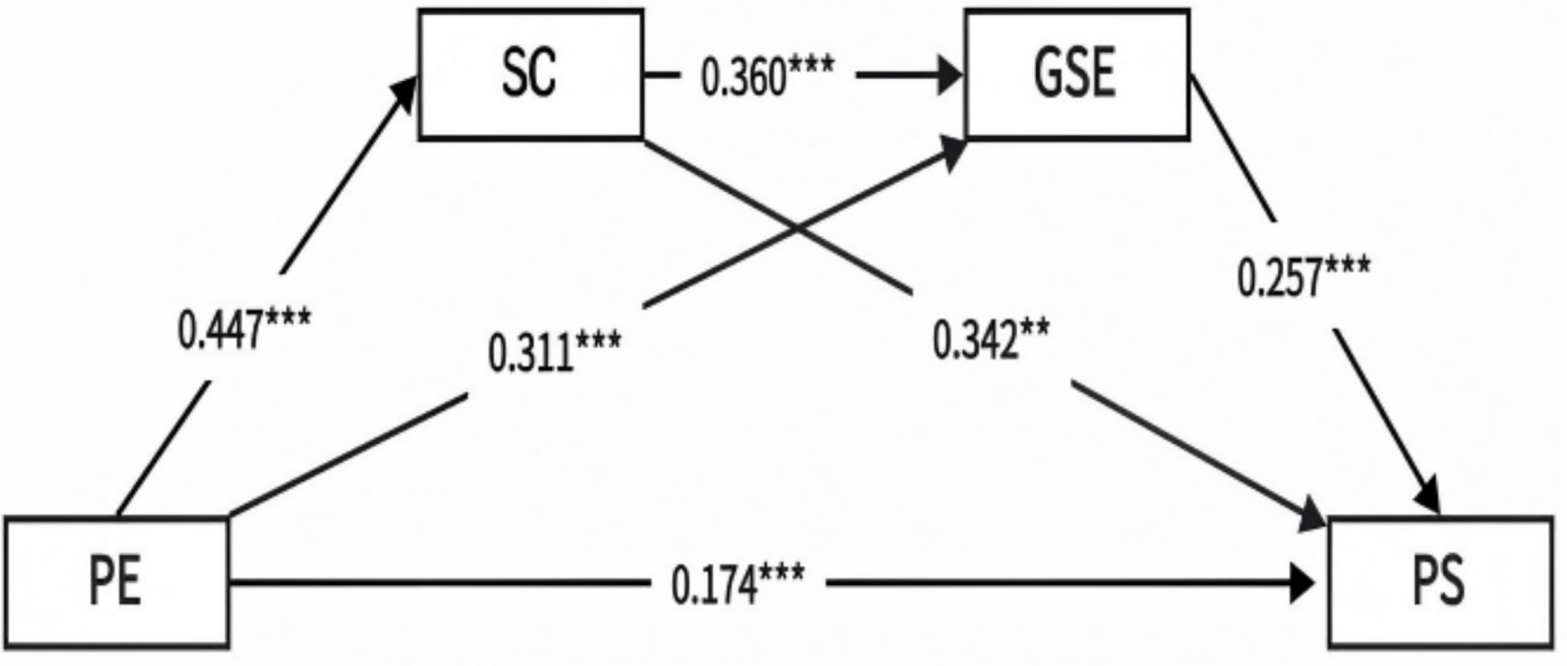
Diagram of the mediation model.

The path coefficients are shown in Figure 2. Using the Bootstrap test with 5,000 repeated samplings, the effect values and confidence intervals of the four paths from physical exercise to prosocial behavior were tested, respectively, (Table 5). The results showed that the total effect of physical exercise on the prosocial behavior of college students was *β* = 0.023, and the 95% CI did not contain 0, indicating that the total effect was significant and Hypothesis 1 was supported. The path coefficient of the direct effect PE → PB was *β* = 0.008, and the 95% CI did not contain 0, indicating that the direct effect of physical exercise on the prosocial behavior of college students was significant. There were three mediation paths in total: Ind1: The mediating role of social conscientiousness in the relationship between physical exercise and prosocial behavior. The mediation effect value was *β* = 0.009, accounting for 38.9% of the total effect, and the 95% confidence interval did not contain 0, indicating that the mediation effect of social conscientiousness was significant. Ind2: The mediating role of general self-efficacy in the relationship between physical exercise and prosocial behavior. The mediation effect value was *β* = 0.004, accounting for 16.9% of the total effect, and the 95% confidence interval did not contain 0, indicating that the mediation effect of general self-efficacy was significant. Ind3: The chain mediating role of social conscientiousness and general self-efficacy in the relationship between physical exercise and the prosocial behavior of college students. The mediation effect value was *β* = 0.002, accounting for 10.4% of the total effect, and the 95% confidence interval did not contain 0, indicating a significant chain mediation effect. The above results indicate that Hypothesis 2, Hypothesis 3, and Hypothesis 4 are supported.

**Table 5:** Mediation effect analysis.

Parameter	Effect value	Product of coef.		Bias-corrected method			Percentile method		
		SE	*T*	Lower	Upper	*p*	Lower	Upper	*p*
PE → PB	0.008	0.002	4.000	0.004	0.012	0.001	0.004	0.012	0.001
PE → SC → PB	0.009	0.002	4.500	0.006	0.013	0.001	0.005	0.013	0.001
PE → GSE → PB	0.004	0.001	4.000	0.002	0.006	0.001	0.002	0.006	0.001
PE → SC → GSE → PB	0.002	0.001	2.000	0.001	0.004	0.001	0.001	0.004	0.001
Total	0.023	0.002	11.500	0.019	0.028	0.001	0.019	0.028	0.001
r1	0.389	0.072	5.403	0.265	0.551	0.001	0.252	0.536	0.001
r2	0.169	0.048	3.521	0.084	0.272	0.001	0.081	0.270	0.001
r3	0.104	0.032	3.250	0.049	0.174	0.001	0.048	0.172	0.001
diff1	0.005	0.002	2.500	0.001	0.010	0.026	0.000	0.010	0.033
diff2	0.007	0.002	3.500	0.003	0.011	0.001	0.003	0.011	0.001
diff3	0.001	0.001	1.000	0.000	0.004	0.043	0.000	0.003	0.067

According to the data analysis, the standardized indirect effects of the three mediation paths Ind1, Ind2, and Ind3 are 0.153, 0.080, and 0.041, respectively. By placing the independent variable, the mediating variable, and the dependent variable on the same standard scale, the path coefficient (*β*) is obtained, which reflects the actual magnitude of the mediation effect (Gu et al., 2024). It can be seen that as a mediating influencing factor, social conscientiousness has the largest path effect coefficient, while the chain mediation path is relatively small. Combining with the analysis of the coefficient of determination and *R*^2^, of each regression equation (SC: 0.200, GSE: 0.327, PS: 0.396), it is concluded that both the mediating variables and the outcome variables in the research model have good explanatory power.

## 5 Discussion

### 5.1 Physical exercise has a significant positive impact on college students’ prosocial behavior

This study found a significant positive correlation between physical exercise and prosocial behavior among college students (*r* = 0.447, *p* < 0.01), with a total effect value of 0.023 (95% CI: 0.019–0.028), which is an above-medium effect level in behavioral science research, indicating that the association between physical exercise and prosocial behavior of college students has practical significance. This result is consistent with existing research findings (Guo, L. R. et al., 2025; Jiang and Bian, 2025), further supporting the effect of physical exercise on social development. From a mechanistic perspective, physical exercise may be associated with prosocial behavior through multiple pathways. Physiologically, regular physical exercise can promote the release of oxytocin and endorphins. These neurotransmitters are related to trust, empathy, and emotional regulation, providing an emotional basis for prosocial behavior (Toresdahl et al., 2024). Psychologically, physical exercise cultivates students’ sense of responsibility and collective honor through goal-setting and rule-following, enabling individuals to better understand others’ situations and be more willing to help others solve problems (Jiang and Bian, 2025). In addition, in the gender subgroup analysis, the physical exercise level of male students was higher than that of female students, but there was no significant difference in prosocial behavior. This may be related to the differences in social expectations, psychological mechanisms, and behavioral motivations between male and female students, which is basically consistent with previous research results (Jiang and Bian, 2025). Notably, the direct effect between physical exercise and prosocial behavior of college students was *β* = 0.174, *p* < 0.001, which remained significant after adding the mediating variables, indicating that there are potential mediating factors not included in the model, such as peer relationships (Wan et al., 2021; Tang et al., 2025), empathy (Shi et al., 2026), emotional intelligence, and motivation for physical education learning (Hui et al., 2022).

### 5.2 The mediating role of social conscientiousness between physical exercise and college students’ prosocial behavior

The research results show that the indirect effect value of Path 1 (physical exercise → social conscientiousness → prosocial behavior) is 0.009, accounting for 38.9% of the total effect. This indicates that social conscientiousness plays an intermediary role between physical exercise and college students’ prosocial behavior. This result is basically consistent with the previous research results (García-García et al., 2023; Guo, L. R. et al., 2025), further supporting the feedback mechanism of “behavior → personality → behavior” in social cognitive theory. As a socialized practice field featuring rules, fairness, and competition, physical exercise gradually strengthens the exercisers’ sense of responsibility, fairness, and collective honor through role division and close cooperation. People with high social conscientiousness pay more attention to image expectations and commitment fulfillment in interpersonal interactions, which provides favorable conditions for prosocial behavior. Therefore, the formation of behavioral habits shapes personality traits, and personality traits in turn determine behavioral outcomes (Feng and Xu, 2026b). It is worth noting that the mediating effect of social conscientiousness in this study is higher than that of self-efficacy, indicating that in the path of the influence of physical exercise on college students’ prosocial behavior, the shaping of personality traits has a greater conduction advantage than cognitive beliefs. This finding is slightly different from the research of (García-García et al., 2023), which may be affected by differences in regional culture, gender ratio, and social expectations.

### 5.3 The mediating role of self-efficacy between physical exercise and prosocial behavior among college students

The data analysis results show that the indirect effect value of Path 2 (physical exercise → general self-efficacy → prosocial behavior) is 0.004, accounting for 16.9% of the total effect value. General self-efficacy plays a partial mediating role between physical exercise and college students’ prosocial behavior. This is basically consistent with the previous research results (Li et al., 2022; Zhan and You, 2024). Analyzing from the mechanism of action, regular physical exercise can not only relieve the life stress of college students and reduce anxiety but also increase their goal-planning ability and behavioral self-confidence, thereby enhancing self-efficacy. College students with high self-efficacy are more likely to show prosocial behavior, which is related to their social cognition and behavioral confidence levels. Specifically, physical exercise may affect general self-efficacy through two paths: physiological and psychological. On the one hand, physical exercise enhances individual functions and behavioral control ability, thus improving general self-efficacy. On the other hand, physical exercise strengthens individuals’ planning and reachability of things in social interaction and goal achievement, which in turn affects the general self-efficacy of college students. In turn, the improvement of self-efficacy not only enhances the effect of physical exercise but also leads to altruistic behaviors such as helping others and teamwork (Zhou et al., 2022). However, the proportion of this path effect is relatively small, indicating that the transmission efficacy of self-efficacy between the two is limited, and there may be more explanatory mediating variables. This result is consistent with the findings of (Jiang and Bian, 2025b).

### 5.4 The chained mediating effect of social conscientiousness and general self-efficacy between college students’ physical exercise and prosocial behavior

A chain mediation analysis revealed that the indirect effect value of the path PE → SC → GSE → PS was 0.002, accounting for 10.4% of the total effect value, which confirmed Hypothesis 4. At the theoretical level, it uncovered the synergistic action path between personality traits and cognitive beliefs, consistent with previous research findings (Hui et al., 2025). The theoretical foundation of this path can be traced back to the “triadic reciprocal determinism” under the social cognitive theory: an individual’s behavior, personality, and cognition interact with each other, jointly shaping social behavior (Luo and Qu, 2025). Individuals with high social conscientiousness are more likely to gain successful experiences and enhance their general self-efficacy when facing social situations due to their stronger planning and self-control abilities (Ma et al., 2024). Moreover, individuals with high self-efficacy are confident in their problem-solving abilities and are more inclined to engage in prosocial behaviors such as active helping and cooperation in interpersonal interactions (Jiang et al., 2025). However, an analysis of the research results indicated that the effect of the chain mediation path was relatively small, accounting for approximately 10% of the total effect. Possible reasons for this may include: First, there was a strong direct correlation between social conscientiousness and general self-efficacy (r = 0.499); Second, there might be effective variables in the path from physical exercise to prosocial behavior that were not included in the research model; Third, the chain mediation required sequential transmission, and the long path might have gradually diluted the effect. In conclusion, the feedback mechanism of “personality → cognition → behavior” revealed by this path has important implications for college physical education. In physical education, teachers should not only focus on teaching sports skills but also reasonably guide students’ sense of responsibility and goal setting to cultivate their sense of responsibility and self-confidence, thereby promoting the development of their prosocial behaviors.

## 6 Conclusion

Based on the data analysis, the following conclusions can be drawn: (1) There are significant correlations among physical exercise, prosocial behavior, social conscientiousness, and general self-efficacy. (2) Physical exercise can significantly and positively predict the prosocial behavior of college students. (3) Physical exercise indirectly predicts the prosocial behavior of college students through social conscientiousness and general self-efficacy, including the single mediating effects of social conscientiousness and general self-efficacy and the chain mediating effect between the two.

## 7 Limitations and future directions

This study reveals the promoting effect of physical exercise on college students’ prosocial behaviors and its underlying mechanism, yet it still has certain limitations. Firstly, the survey samples are mainly concentrated among college students in Hubei and Henan regions. The similarity in economic level and regional culture endows the sample representativeness with obvious regional characteristics. In the future, it is necessary to further expand the sample size in the eastern and western regions to enhance the generality and representativeness of the study. Secondly, this study is a cross-sectional study, making it difficult to clarify the causal relationships among various factors. It can only prove the existence of correlations between variables, and the causal consideration of time series cannot be incorporated into the analysis and discussion, resulting in certain limitations in the analysis. In the future, more scientific and reasonable explanations need to be obtained through longitudinal tracking and experimental intervention. Finally, the measurement method in this study mainly relies on self-report, which may be affected by individual cognitive biases and social norms, leading to collective biases in the reliability and validity of the data. In the future, it is necessary to combine multiple survey methods such as behavioral observation and instrumental measurement to improve the objectivity, reliability, and validity of the sample data.

## Data Availability

The original contributions presented in the study are included in the article/supplementary material, further inquiries can be directed to the corresponding author.
